# Prevalence of attention deficit hyperactivity disorder symptoms in patients with schizophrenia

**DOI:** 10.1111/acps.12948

**Published:** 2018-08-13

**Authors:** I. Arican, N. Bass, K. Neelam, K. Wolfe, A. McQuillin, G. Giaroli

**Affiliations:** ^1^ Molecular Psychiatry Laboratory Division of Psychiatry University College London London UK; ^2^ Greater Manchester Mental Health NHS Foundation Trust Bolton UK

**Keywords:** cognitive impairment, comorbidity, psychoses, diagnosis, ADHD

## Abstract

**Objective:**

To investigate the frequency of childhood and adult attention deficit hyperactivity disorder (ADHD) symptoms in a cohort of patients with schizophrenia (SCZ).

**Methods:**

A systematic review was conducted to evaluate existing evidence. Two self‐report questionnaires were used to investigate adult ADHD and childhood ADHD symptoms in 126 patients with ICD‐10 diagnoses of SCZ.

**Results:**

Five studies were included in the systematic review, with the prevalence of childhood and adult ADHD in SCZ subjects ranging between 17–57% and 10–47% respectively. Within our cohort, 47% of patients reported positive screening for ADHD symptoms either in childhood or adulthood. 23% reported symptomatology consistent with both childhood and adult ADHD.

**Conclusions:**

We demonstrate a greater presence of ADHD symptomatology in SCZ compared to that reported for ADHD in the general population. Our findings highlight the importance of improved clinical assessment and treatment considerations in a subgroup of patients with SCZ.

**Significant outcomes**

- 23% of patients with schizophrenia report childhood and adult attention deficit hyperactivity disorder symptoms compared to 2.5% of adults diagnosed in the general population.

**Limitations**

- The questionnaires are not diagnostic.
- Patients in the sample were not screened for active SCZ or mood symptoms.
- The lack of a new control group from the general population for comparison purposes.

## Introduction

Schizophrenia (SCZ) and attention deficit hyperactivity disorder (ADHD) are both neurodevelopmental disorders. SCZ is defined by the presence of psychotic symptoms, disorganised speech/behaviour and negative symptoms, such as avolition and alogia 1. However, neurocognitive deficits are increasingly recognised as a core feature of SCZ, associated with functional outcomes 2 and quality of life 3, 4. SCZ has a population prevalence of approximately 1% 5, with a male‐to‐female ratio of 1.4 : 1 6. ADHD is a behavioural disorder characterised by pervasive and impairing symptoms of inattention, hyperactivity and impulsivity before the age of 12 7. The prevalence in children aged 18 or younger is estimated to be 5.29% 8, with a male‐to‐female ratio of 4 : 1 in the general population 9. Diagnostic follow‐up studies have confirmed ADHD can persist into adulthood with an estimated prevalence of 2.5% in the adult population 10. Furthermore, the symptoms of ADHD in childhood and their persistence into adulthood have been reported to be 10.1% and 4.6%, respectively, in a general adult population 11.

The presentation of SCZ can include varying degrees of attentional dysfunction 12, 13, a central cognitive deficit observed in patients with SCZ 2. Recent work has revealed a genetic correlation between SCZ and ADHD, meaning that there is some overlap between the risk alleles for SCZ and for ADHD 14. Specifically, the genetic correlation (rg) between the two disorders has been estimated to be 0.122 (*P* = 0.0007) 15. Prodromal attention deficit symptoms have retrospectively been identified in patients with SCZ 16, and a prospective study in a Danish cohort has shown that a diagnosis of ADHD is a strong risk factor (RR 4.3) for the development of SCZ 17. Additionally, an odds ratio of 6.7 (95% CI 5.9–7.5) for probands with ADHD to develop SCZ was reported in a Swedish cohort 18. Furthermore, the most common comorbid condition found in childhood‐onset SCZ is ADHD, at 84% 19. Thus, ADHD‐like features have been suggested to be an early vulnerability marker for SCZ 20.

### Aims of the study

We aimed to:

- Systematically review studies that have estimated the prevalence of childhood and adult ADHD in patients with schizophrenia
- Estimate the prevalence of childhood and adult attention deficit hyperactivity disorder symptoms in a cohort of patients diagnosed with schizophrenia (UCL genetics of schizophrenia cohort).

## Methods

### Systematic review of prevalence of ADHD in people with schizophrenia

We systematically reviewed the literature relating to the prevalence of childhood ADHD (cADHD) and the persistence in adulthood of the condition (c+aADHD) in patients with SCZ using the PRISMA criteria 21. Eligible studies included patients with a SCZ spectrum disorder as defined by DSM‐IV or DSM‐V or ICD‐10 (the 10^th^ revision of the International Statistical Classification of Disease and Related Health Problems) 22.

MEDLINE and PsycINFO were searched via OvidSP using the terms: ([Attention deficit hyperactivity disorder] OR [ADHD] AND [Schizophrenia] AND [Prevalence]) on, 29 May 2018. A manual search of the literature was also conducted. Only studies reported in English were considered for the review. Study selection was performed by two reviewers (IA and GG).

The outcome measures were the percentage of patients with SCZ that had a clinical diagnosis of ADHD or ADHD symptoms, as determined by interviews and self‐report. A data extraction table capturing the relevant information was developed. Data items extracted included: the number of trial participants, the age range of participants, method of diagnosis of trial participants, methods for assessing the occurrence of ADHD symptoms in trial participants and the outcome measure of prevalence.

### Estimation of prevalence of ADHD symptoms in UCL genetics of schizophrenia cohort

#### Participants

The SCZ cohort of the UCL DNA Polymorphisms in Mental Illness (DPIM) has been described previously 23. Briefly, participants were recruited from NHS services. Potential participants with an ICD‐10 diagnosis of schizophrenia were interviewed using the Schedule for Affective Disorders and Schizophrenia‐Lifetime version (SADS‐L) 24, and the 90‐item Operational Criteria Checklist (OPCRIT) was also completed 25. Participants who achieved a Research Diagnostic Criteria (RDC) 26 diagnosis of schizophrenia with the SADS‐L were included. Ethical approval was granted for the study by the NHS National Research Ethics Service, NRES Committee South Central – Southampton A (formally the Metropolitan Multi‐centre Research Ethics Committee) REC Reference Number: 03/11/090. Written informed consent was obtained from each participant. For the SCZ and attention deficit and hyperactivity symptom study described here, 1000 participants from the SCZ cohort of the UCL DPIM study were invited to participate by letter.

#### Assessment of ADHD symptoms

Validated tools were used for the assessment of cADHD symptoms and adult ADHD symptoms (aADHD). The 25‐item version of the Wender Utah Rating Scale (WURS) provided information about symptomatology consistent with cADHD based on DSM criteria 27. The part‐A screening test from the Adult ADHD Self‐Report Scale (ASRS) was utilised to identify the presence of symptoms consistent with aADHD 28.

OPCRIT and/or SADS‐L data were available for 106 of 126 of the participants with SCZ. The earliest age when medical advice was sought or the age of onset of first psychiatric condition (AOO), the age at study entry (ASE), the presence of cannabis abuse/dependence, the presence of alcohol abuse/dependence, the presence of any abuse/dependence (cannabis/alcohol/other), a family history of SCZ, a family history of other psychiatric condition and the length of schooling were extracted from the phenotypic data and used as covariates.

### Statistical analysis

Stata (version 14) was used for all statistical analyses 29. Data cleaning was performed. Responses were checked for missing or inconsistent data. Participants who returned more than three missing answers on the WURS or more than one on the ASRS were excluded.

The total scores for the WURS and ASRS were calculated for each participant. Data imputation was performed using the subject's average score for the questionnaire affected. A suitable cut‐off for each scale was applied to dichotomise the data for analysis. As there was no evidence for the best WURS cut‐off score in patients with SCZ, we used a cut‐off at 46 of 100 due to its greater discriminatory power in patients with unipolar depression 27.

The ASRS has a maximum score of 30 (six questions with maximum score of 5). Each question has a different ‘cut‐off’ for what is classified as a significant symptom, so for one question it might be 3/5 and for the other 4/5. If a person achieves the cut‐off in four of the six questions, they are considered to have symptoms that are highly consistent with ADHD in adults, warranting further clinical investigation 28. In order to implement a questionnaire‐wide cut‐off score, each question was coded to either 1 or 0 depending on whether its cut‐off was reached. Scores of 4 and above were considered positive, and less than 4 were considered to be negative.

To compare the means of continuous variables, we utilised either the t‐test or the nonparametric Mann–Whitney *U*‐test following assessment for normality. A one‐way analysis of variance was used to compare groups of participants with different lifetime prevalences of ADHD symptomatology for AOO and ASE. Linear regression of these covariates with WURS and ASRS scores was also carried out to confirm the association.

The Mann–Whitney *U*‐test was utilised to compare whether scoring of each questionnaire was affected by different covariates. To confirm these findings, linear regression was carried out on those covariates that produced indicative significance at the 5% level.

## Results

### Systematic review findings

The systematic search identified 299 studies. After removal of duplicates, and filtering to English‐only studies, 226 studies remained. Title and abstract screening identified 10 studies for detailed review. Five articles fulfilled our inclusion criteria, three following the above literature search and 2 from a manual search of databases and reference lists of articles.

Prevalence estimates for cADHD in patients affected by SCZ ranged from 17 to 57%. Only two studies measured the prevalence of ADHD comorbidity in adulthood, reporting 10% and 47%, respectively, see Table 1 30, 31. Table 2 details the study characteristics of each eligible study.

**Table 1:** Prevalence of cADHD and/or aADHD reported in the current literature for patients with SCZ

Author/Source		Reported prevalence	Sample (*N*)
Peralta et al. (2011) 33		17% cADHD	112
Dalteg et al. (2014) 31	Sample 1	57% cADHD 47% cADHD + aADHD	119
	Sample 2 + 3	50% cADHD	247
Rubino et al. (2009) 34		42.6% cADHD	197
Hallerbäck et al. (2014) 30		10% cADHD + aADHD	41
Donev et al. (2011) 32		44.4% cADHD	27

cADHD, childhood ADHD; aADHD, adulthood ADHD.

**Table 2:** The study characteristics of the five eligible studies included in the systematic review

Source	No. of patients (*N*)		Age range	Inclusion criteria	Exclusion criteria	Diagnostic tools
Peralta et al. (2011) 33	112		15–65 years	Patients admitted for treatment of first episode of psychosis and met a DSM‐IV diagnosis of schizophrenia, schizophreniform or schizoaffective disorder. Patients had no previous exposition to antipsychotics and an available biological mother.	A history of drug dependence, IQ < 70, evidence of organic brain disorder or any other meaningful medical illness.	An 18‐item checklist that included DSM‐IV criteria for ADHD was completed by mothers, regarding the ages of 6–10 years. This was then reviewed by researchers who also enquired about age of onset, severity and prior ADHD diagnoses
Dalteg et al. (2014) 31	Gotland Sample 1 inpatient ward	119	16–68 years	In‐patients who met DSM‐IV criteria for SCZ	The samples excluded drug‐induced psychoses	Subjects were interviewed by experienced psychologist and a clinical researcher about childhood symptoms. The self‐report Malmo questionnaire assessed adult ADHD (includes the 18 DSM‐IV criteria)
	Aftercare Sample 2 forensic	149	20–75 years	Men with a DSM‐IV‐TR SCZ diagnosis were recruited from forensic psychiatric clinics when released from compulsory care.		All patients were assessed by experienced and trained interviewers. Retrospective cADHD diagnosis was based on the presence of a typical pattern of problems (at time of data collection, there was no ADHD SCID interview)
	Aftercare Sample 3 ‐general clinics	98	19–63 years	Control patients were of the same age, sex and released from general psychiatry clinics near Sample 2 clinics and had a DSM‐IV‐TR SCZ diagnosis		
Rubino et al. (2009) 34	197		35.5 mean (SD = 11.1)	In‐patients with DSM‐IV diagnoses of adult‐onset SCZ	Schizophreniform, schizoaffective and delusional disorders were excluded, patients scoring less than 24 on the Mini Mental State Examination and with clinical evidence of brain damage/disease	Two senior psychiatrists employed the SCID interview for current diagnosis. The semistructured interview K‐SADS‐PL was used to retrospectively identify Axis I disorders in childhood and adolescence
Hallerbäck et al. (2014) 30	41		28.9 mean (SD = 4.6)	All participants provided informed consent and were recruited from the only adult psychiatric clinic in Värmland, Sweden.	Their clinical diagnosis had to be confirmed by the SCID‐I, or participants were excluded	With a SCID interview, participants were asked about prior diagnostic assessments, and specifically whether they had been examined for, or had a clinical diagnosis of ADHD
Donev et al. (2011) 32	27		18–44 years	All patients had a schizophrenic disorder according to ICD‐10 criteria. Informed consent was obtained, and tests were carried out a few days before discharge (likely to be in remission)	Exclusion criteria included any severe psychotic symptoms, medication‐induced psychomotor retardation, severe schizophrenic residual symptoms or a comorbid affective disorder	The 25‐item version of the WURS was used to measure ADHD, with a cut‐off at 30

### Prevalence of lifetime ADHD symptoms in UCL genetics of schizophrenia cohort

Consent‐to‐participate forms were received from 194 of the 1000 participants invited to take part. Questionnaires were received from 131 subjects; 5 (3.8%) had to be excluded due to the level of missing data leaving a final sample size of 126 (83 (65.9%) men and 43 (34.1%) women). Of these participants, 10 (7.9%) required imputation for one value, 9 (7.1%) for two values and 3 (2.4%) for three missing values. Participants included in the study were aged 21–85.

Twenty‐three per cent of participants with SCZ reported symptoms of ADHD in childhood persisting through to adulthood. Symptoms of cADHD only were reported by 11.1%, and aADHD only by 12.7%. The remaining 53.2% of participants reported no ADHD symptoms. In total, 34.1% of 126 subjects with SCZ reported symptomatology consistent with childhood ADHD. Table 3 shows the breakdown of each category for ADHD symptomatology.

**Table 3:** Prevalence of cADHD and/or aADHD symptomatology reported by patients with SCZ in the UCL genetics of schizophrenia cohort

Lifetime ADHD symptomatology	Freq: (*N* = 126)	Per cent (%)	Age range	Gender *N* (%) male
c+aADHD	29	23.0	23–77	19 (65.5)
aADHD only	16	12.7	35–70	10 (62.5)
cADHD only	14	11.1	29–65	9 (64.3)
No ADHD	67	53.2	21–85	45 (67.2)

cADHD, childhood ADHD symptomatology; aADHD, adulthood ADHD symptomatology; c+aADHD, child and adulthood ADHD symptomatology.

As part of the phenotypic data set collected for the DPIM study, information on the AOO was available for 106 participants and ASE for 99. A one‐way analysis of variance revealed no significant difference in the AOO (*P* = 0.60) or ASE (*P* = 0.40) in any of the four ADHD symptom groups (cADHD, aADHD, c+aADHD and no ADHD). A linear regression of AOO with ASRS or WURS scores also showed no significance (*P* < 0.05).

### Secondary analyses

OPCRIT and SADS‐L variables were selected to examine their influence on mean WURS and ASRS scores, as shown in Table 4.

**Table 4:** Mean WURS and ASRS scores with the presence of covariates in the UCL genetics of schizophrenia cohort

Covariate	*N*	WURS		ASRS	
		Mean score	Analyses	Mean score	Analyses
History of alcohol dependence: Y/N	101	Y: 47.6 (6.8) N: 34.8 (2.4)	*U* = 11555.7 *P* = 0.062	Y: 20.7 (1.6) N: 16.4 (0.5)	*U* = 11514.6 *P* = 0.015*
History of cannabis dependence: Y/N	71	Y: 41.5 (8.0) N: 37.6 (3.0)	*U* = 3022.4 *P* = 0.48	Y: 19.8 (2.4) N: 17.4 (0.7)	*U* = 3012.3 *P* = 0.26
History of any abuse/dependence: Y/N	106	Y: 48.3 (5.5) N: 32.8 (2.3)	*U* = 14730.9 *P* = 0.0048*	Y: 20.6 (1.3) N: 16.5 (0.5)	*U* = 14673.3 *P* = 0.0043*
Education‐A high school graduate: Y/N	73	Y: 35.5 (2.9) N: 46.4 (7.6)	*U* = 5091.2 *P* = 0.14	Y: 17.5 (0.7) N: 18.4 (1.8)	*U* = 5069.0 *P* = 0.47

WURS, Wender Utah Rating Scale; ASRS, Adult ADHD Self‐Report Scale; Y/N Yes/No; *: Significant at 5% level, statistical comparison utilised the Mann–Whitney *U*‐test.

Univariate linear regression analyses were carried out for variables with significant associations at the 5% level in Table 4. Regression for the history of any substance abuse with WURS (*P* = 0.0071) and ASRS (*P* = 0.0015) was significant, and a linear regression of ASRS scores on a history of alcohol abuse showed a significant association, *F* (1, 99) = 8.99, *P* = 0.0034.

## Discussion

A systematic review of the published literature of ADHD symptomatology in schizophrenia patients identified only five previous studies, with small sample sizes and heterogeneous study methodologies. For example, Hallerbäck et al. and Donev et al. had a sample size of 41 and 27 patients respectively 30, 32. There were also differences between studies in the criteria used to define ADHD. For example, Dalteg et al. 31 defined ADHD by a ‘typical pattern of problems’ and Donev et al. 32 adopted a less stringent cut‐off value for the WURS. The studies also varied in the ascertainment method of schizophrenia patients. For example, Peralta et al. (2011) included patients suffering from first episode SCZ spectrum psychosis 33, Rubino et al. (2009) included in‐patients with adult‐onset SCZ 34, and Hallerbäck et al. (2014) included patients ascertained from an adult psychiatric clinic 30. The majority of studies only assessed cADHD symptoms in patients with SCZ and therefore were susceptible to recall bias. Only two studies investigated the prevalence of ADHD comorbidity. The ADHD symptom prevalence results from our study fall in the midrange for prevalence of both c+aADHD and cADHD reported in the literature for people suffering from SCZ (Table 1).

In our cohort, we found 23% of participants with SCZ to have symptomatology consistent with c+aADHD, increasing to 34.1% when including cADHD only. Despite the heterogeneity in the studies of ADHD in SCZ, all estimates of ADHD prevalence and our estimate of symptomatology indicate that ADHD is more common in SCZ than in the general population. The general population prevalence of ADHD diagnosis has been estimated to be 5.3% in childhood and 2.5% in adults 8, 10. This rises to 10.1% for cADHD and 4.6% for c+aADHD in a general population sample from Germany (limited to 18‐ to 64‐year‐olds) where the WURS and ASRS were used to estimate prevalence 11. In our study, 35.7% of participants reported ADHD symptoms in adulthood, and this is higher than reported estimates of adult ADHD symptomatology in the general population of 5.7–10.9% obtained using the ASRS 35, 36, 37.

Current diagnostic criteria for ADHD require the onset of symptoms in childhood, that is, before the age of 12 in DSM‐5 and before the age of 7 according to the ICD‐10. Interestingly, although 35.7% of participants with SCZ reported aADHD symptoms, 12.7% did not report symptomatology suggestive of cADHD. Longitudinal studies of epidemiological cohorts in Brazil 38, UK 39 and New Zealand 40 have recently reported the presence of adult‐onset ADHD in the absence of childhood‐onset ADHD. This provides support for a model whereby adult‐onset ADHD is not necessarily a continuation of childhood‐onset ADHD, suggesting that there may be distinct developmental trajectories. It remains unclear whether adult‐onset ADHD is a separate diagnostic entity or whether it is an artefact of misdiagnosis or overlapping criteria (methodological bias). Ideally, a diagnosis in an adult is a process that should include a retrospective analysis of the ADHD symptoms in childhood. We find the same results as the longitudinal epidemiological ADHD studies, whereby only a proportion of patients with SCZ meet the criteria for adult‐onset ADHD and this would be an important group to study in further research.

Despite AOO being conceptualised as a surrogate measure of severity 41, and an earlier age of onset being associated with more severe cognitive impairments in SCZ 42, our results did not suggest a significant association between age of onset of SCZ and ADHD symptoms. There was also no evidence that the age at which people entered the study influenced their childhood or adult ADHD scores suggesting that this was not a major factor that influenced recall of symptoms. In line with the literature, higher ADHD scores were predictive of a history of any abuse/dependence 43 and were implicated in poorer outcome and treatment compliance in SCZ 44. Also in line with the literature are the findings that participants reporting aADHD symptoms are more vulnerable to alcohol abuse 45. Thus, diagnosing comorbidities in clinical practice could help to identify those most at risk of poorer outcomes and improve interventions and support.

There is currently a lack of recommended pharmacological treatments for people experiencing comorbid SCZ and ADHD 46. SCZ is primarily treated with dopamine D2‐antagonists or partial agonists that are often classed as typical or atypical antipsychotics. ADHD in contrast is treated with ‘dopamine enhancers’, through blockers of the presynaptic dopamine transporter (DAT) and/or noradrenalin transporters 47. Furthermore, patients with comorbid ADHD fail to respond to antipsychotics for their SCZ have poorer outcomes 48 and are vulnerable to the lack of treatment focus on the cognitive impairments seen in patients with SCZ 49. Reports have suggested that psychostimulants typically used in ADHD can alleviate the negative symptoms 50 and cognitive deficits 51 of SCZ; however, there is evidence showing their potential dangers in triggering psychotic symptoms, leaving room for further debate 52, 53. None of the participants in our study reported being prescribed psychostimulants.

This study has multiple limitations which must be acknowledged. First, the sample size was modest, but is similar to comparable studies in the literature. More precise estimates of the prevalence of cADHD and/or aADHD in SCZ will require much larger sample sizes. Second, the sample comprised of participants who volunteered to take part in the DPIM study and responded to the invitation to take part in the study extension exploring the prevalence of inattentiveness, hyperactivity or impulsiveness in people living with schizophrenia. The response rate was below 20%. Moreover, the participants had to complete and return two letters. These factors likely biased the cohort towards people with higher levels of functioning and may impact the applicability of the study data to other settings.

Third, the gold standard assessment for ADHD in adults is the Diagnostic Interview for ADHD in Adults (DIVA) 54; however, we did not specifically aim to diagnose ADHD in our cohort but aimed to assess ADHD symptomology as a measurement of a continuous variable. Furthermore, we were not able to conduct face‐to‐face interviews. Fourth, we did not have a measure of current SCZ symptomology when ADHD symptoms were assessed. Additionally, current mood symptoms were not assessed, which may also have had an effect on ADHD symptom scores, and the WURS is vulnerable to recall bias. Another limitation is that the ASRS has not been validated in patients with SCZ. This might lead to the risk of methodical overlap between symptoms that are more relevant to ADHD with those that are more relevant to SCZ or to functional cognitive impairment. Moreover, we opted to include only part A of the ASRS which has been shown to have a positive predictive value of up to 94.7 at detecting clinical cases of adult ADHD 28. Our decision was guided by the aim of keeping our questionnaire items to the lowest possible number, in order to maximise responses. By doing so, however, we recognise the reduced ability to perform ADHD subtyping. Future work using the full 18‐item ASRS in participants suffering from SCZ has the potential to reveal interesting and important insight into subtypes of ADHD that are observed in SCZ.

We report a higher prevalence of ADHD symptoms in patients with SCZ, as compared to the prevalence of ADHD symptoms within the general population. These findings likely reflect a degree of overlapping symptomatology for the two disorders and also some of the shared genetic aetiology that has been identified in recent genomewide association studies 15. The importance of these findings lies in the contrasting therapies recommended for each condition.

## Declaration of interest

Dr Giovanni Giaroli has received honoraria for acting as a consultant and speaker and for travelling by Shire, Eli Lilly, Flynn Pharma and Janssen.

## References

[1] American Psychiatric Association . Diagnostic and statistical manual of mental disorders (5th edn). Arlington, VA: American Psychiatric Association; 2013. 20, 31‐32, 87‐88, 100‐104, 155‐165.

[2] Green MF . What are the functional consequences of neurocognitive deficits in schizophrenia? Am J Psychiatry 1996;153:321–330. 8610818 10.1176/ajp.153.3.321

[3] Savilla K , Kettler L , Galletly C . Relationships between cognitive deficits, symptoms and quality of life in schizophrenia. Aust New Zeal J Psychiatry 2008;42:496–504. 10.1080/00048670802050512 18465376

[4] Ueoka Y , Tomotake M , Tanaka T et al. Quality of life and cognitive dysfunction in people with schizophrenia. Prog Neuro‐Psychopharmacol Biol Psychiatry 2011;35:53–59. 10.1016/j.pnpbp.2010.08.018 20804809

[5] Carpenter WT , Buchanan RW . Schizophrenia. N Engl J Med 1994;330:681–690. 8107719 10.1056/NEJM199403103301006

[6] McGrath J , Saha S , Chant D , Welham J . Schizophrenia: a concise overview of incidence, prevalence, and mortality. Epidemiol Rev 2008;30:67–76. 18480098 10.1093/epirev/mxn001

[7] Epstein JN , Loren REA . Changes in the definition of ADHD in DSM‐5: subtle but important. Neuropsychiatry (London) 2013;3:455–458. 24644516 10.2217/npy.13.59 PMC3955126

[8] Polanczyk G , de Lima MS , Horta BL , Biederman J , Rohde LA . The worldwide prevalence of ADHD: a systematic review and metaregression analysis. Am J Psychiatry 2007;164:942–948. 17541055 10.1176/ajp.2007.164.6.942

[9] American Psychiatric Association . Diagnostic and statistical manual of mental disorders (4th edn.). Arlington, VA: American Psychiatric Association 2000; 210: 373–374.

[10] Simon V , Czobor P , Balint S , Meszaros A , Bitter I . Prevalence and correlates of adult attention‐deficit hyperactivity disorder: meta‐analysis. Br J Psychiatry 2009;194:204–211. 19252145 10.1192/bjp.bp.107.048827

[11] Berger NAA , Müller A , Brähler E , Philipsen A , de Zwaan M . Association of symptoms of attention‐deficit/hyperactivity disorder with symptoms of excessive exercising in an adult general population sample. BMC Psychiatry 2014;14:250. 25214027 10.1186/s12888-014-0250-7 PMC4172949

[12] Green MF , Harvey PD . Cognition in schizophrenia: past, present, and future. Schizophr Res Cogn 2014;1:e1–e9. 25254156 10.1016/j.scog.2014.02.001 PMC4171037

[13] Nuechterlein KH , Dawson ME . Information processing and attentional functioning in the developmental course of schizophrenic disorders. Schizophr Bull 1984;10:160–203. 6729409 10.1093/schbul/10.2.160

[14] Hamshere ML , Stergiakouli E , Langley K et al. Shared polygenic contribution between childhood attention‐deficit hyperactivity disorder and adult schizophrenia. Br J Psychiatry 2013;203:107–111. 23703318 10.1192/bjp.bp.112.117432 PMC3730114

[15] Demontis D , Walters RK , Martin J et al. Discovery of the first genome‐wide significant risk loci for ADHD. Nat Genet 2000. 10.1038/s41588-018-0269-7 PMC6481311 30478444

[16] Silverstein ML , Mavrolefteros G , Turnbull A . Premorbid factors in relation to motor, memory, and executive functions deficits in adult schizophrenia. Schizophr Res 2003;61:271–280. 12729879 10.1016/s0920-9964(02)00312-2

[17] Dalsgaard S , Mortensen PB , Frydenberg M , Maibing CM , Nordentoft M , Thomsen PH . Association between attention‐deficit hyperactivity disorder in childhood and schizophrenia later in adulthood. Eur Psychiatry 2014;29:259–263. 24016863 10.1016/j.eurpsy.2013.06.004

[18] Larsson H , Rydén E , Boman M , Långström N , Lichtenstein P , Landén M . Risk of bipolar disorder and schizophrenia in relatives of people with attention‐deficit hyperactivity disorder. Br J Psychiatry 2013;203:103–106. 23703314 10.1192/bjp.bp.112.120808 PMC3730113

[19] Ross RG , Heinlein S , Tregellas H . High rates of comorbidity are found in childhood‐onset schizophrenia. Schizophr Res 2006;88:90–95. 16916600 10.1016/j.schres.2006.07.006

[20] Keshavan MS , Sujata M , Mehra A , Montrose DM , Sweeney JA . Psychosis proneness and ADHD in young relatives of schizophrenia patients. Schizophr Res 2003;59:85–92. 12413647 10.1016/s0920-9964(01)00400-5

[21] Liberati A , Altman DG , Tetzlaff J et al. The PRISMA statement for reporting systematic reviews and meta‐analyses of studies that evaluate healthcare interventions: explanation and elaboration. BMJ 2009;339:b2700. 19622552 10.1136/bmj.b2700 PMC2714672

[22] Brämer GR . International statistical classification of diseases and related health problems. Tenth revision. World Health Stat Q 1988;41:32–36. 3376487

[23] Fiorentino A , Sharp SI , McQuillin A . Association of rare variation in the glutamate receptor gene SLC1A2 with susceptibility to bipolar disorder and schizophrenia. Eur J Hum Genet 2015;23:1200–1206. 25406999 10.1038/ejhg.2014.261 PMC4351899

[24] Endicott J , Spitzer RL . A diagnostic interview: the schedule for affective disorders and schizophrenia. Arch Gen Psychiatry 1978;35:837–844. 678037 10.1001/archpsyc.1978.01770310043002

[25] McGuffin P , Farmer A , Harvey I . A polydiagnostic application of operational criteria in studies of psychotic illness: development and reliability of the OPCRIT system. Arch Gen Psychiatry 1991;48:764–770. 1883262 10.1001/archpsyc.1991.01810320088015

[26] Spitzer RL , Endicott J , Robins E . Research diagnostic criteria: rationale and reliability. Arch Gen Psychiatry 1978;35:773–782. 655775 10.1001/archpsyc.1978.01770300115013

[27] Ward MF , Wender PH , Reimherr FW . The Wender Utah rating scale: an aid in the retrospective diagnosis of childhood attention deficit hyperactivity disorder. Am J Psychiatry 1993;150:885–890. 8494063 10.1176/ajp.150.6.885

[28] Kessler RC , Adler L , Ames M et al. The World Health Organization adult ADHD self‐report scale (ASRS): a short screening scale for use in the general population. Psychol Med 2005;35:245–256. 15841682 10.1017/s0033291704002892

[29] StataCorp . Stata statistical software: release 14. College Station, TX: StataCorp LP; 2015.

[30] Hallerbäck MU , Lugnegard T , Gillberg C . ADHD and nicotine use in schizophrenia or asperger syndrome: a controlled study. J Atten Disord 2014;18:425–433. 22498753 10.1177/1087054712439099

[31] Dalteg A , Zandelin A , Tuninger E , Levander S . Psychosis in adulthood is associated with high rates of ADHD and CD problems during childhood. Nord J Psychiatry 2014;68:560–566. 24620816 10.3109/08039488.2014.892151

[32] Donev R , Gantert D , Alawam K et al. Comorbidity of schizophrenia and adult attention‐ deficit hyperactivity disorder. World J Biol Psychiatry 2011;12:1562–2975. 10.3109/15622975.2011.599212 21905996

[33] Peralta V , de Jalón EG , Campos MS , Zandio M , Sanchez‐Torres A , Cuesta MJ . The meaning of childhood attention‐deficit hyperactivity symptoms in patients with a first‐episode of schizophrenia‐spectrum psychosis. Schizophr Res 2011;126:28–35. 20926260 10.1016/j.schres.2010.09.010

[34] Rubino A , Frank E , Croce Nanni R et al. A comparative study of axis I antecedents before age 18 of unipolar depression, bipolar disorder and schizophrenia. Psychopathology 2009;42:325–332. 19672135 10.1159/000232975

[35] Stickley A , Koyanagi A , Takahashi H , Kamio Y . ADHD symptoms and pain among adults in England. Psychiatry Res 2016;246:326–331. 27750114 10.1016/j.psychres.2016.10.004

[36] Das D , Cherbuin N , Butterworth P , Anstey KJ , Easteal S . A population‐based study of attention deficit/hyperactivity disorder symptoms and associated impairment in middle‐aged adults. PLoS ONE 2012;7:e31500. 22347487 10.1371/journal.pone.0031500 PMC3275565

[37] Alaheino L , Leppämäki S . Prevalence of ADHD symptoms among adults in the general population in Finland. Eur Psychiatry 2017;41:S352.

[38] Caye A , Rocha TB‐M , Anselmi L et al. Attention‐deficit/hyperactivity disorder trajectories from childhood to young adulthood. JAMA Psychiatry 2016;73:705. 27192050 10.1001/jamapsychiatry.2016.0383

[39] Agnew‐Blais JC , Polanczyk GV , Danese A , Wertz J , Moffitt TE , Arseneault L . Evaluation of the persistence, remission, and emergence of attention‐deficit/hyperactivity disorder in young adulthood. JAMA Psychiatry 2016;73:713. 27192174 10.1001/jamapsychiatry.2016.0465 PMC5475268

[40] Moffitt TE , Houts R , Asherson P et al. Is adult ADHD a childhood‐onset neurodevelopmental disorder? Evidence from a four‐decade longitudinal cohort study. Am J Psychiatry 2015;172:967–977. 25998281 10.1176/appi.ajp.2015.14101266 PMC4591104

[41] DeLisi LE . The significance of age of onset for schizophrenia. Schizophr Bull 1992;18:209–215. 1377833 10.1093/schbul/18.2.209

[42] Hoff AL , Harris D , Faustman WO et al. A neuropsychological study of early onset schizophrenia. Schizophr Res 1996;20:21–28. 8794490 10.1016/0920-9964(95)00065-8

[43] Wilens TE , Morrison NR . The intersection of attention‐deficit/hyperactivity disorder and substance abuse. Curr Opin Psychiatry 2011;24:280–285. 21483267 10.1097/YCO.0b013e328345c956 PMC3435098

[44] Winklbaur B , Ebner N , Sachs G , Thau K , Fischer G . Substance abuse in patients with schizophrenia. Dialogues Clin Neurosci 2006;8:37–43. 16640112 10.31887/DCNS.2006.8.1/bwinklbaur PMC3181760

[45] Smith BH , Molina BSG , Pelham WE . The clinically meaningful link between alcohol use and attention deficit hyperactivity disorder. Alcohol Res Heal 2002;26:122–129.

[46] Pallanti S , Salerno L . Raising attention to attention deficit hyperactivity disorder in schizophrenia. World J Psychiatry 2015;5:47–55. 25815254 10.5498/wjp.v5.i1.47 PMC4369549

[47] Dichter GS , Damiano CA , Allen JA . Reward circuitry dysfunction in psychiatric and neurodevelopmental disorders and genetic syndromes: animal models and clinical findings. J Neurodev Disord 2012;4. 10.1186/1866-1955-4-19 PMC3464940 22958744

[48] Elman I , Sigler M , Kronenberg J et al. Characteristics of patients with schizophrenia successive to childhood attention deficit hyperactivity disorder (ADHD). Isr J Psychiatry Relat Sci 1998;35:280–286. 9988985

[49] Marder SR , Fenton W . Measurement and treatment research to improve cognition in schizophrenia: NIMH MATRICS initiative to support the development of agents for improving cognition in schizophrenia. Schizophr Res 2004;72:5–9. 15531402 10.1016/j.schres.2004.09.010

[50] Lindenmayer J‐P , Nasrallah H , Pucci M , James S , Citrome L . A systematic review of psychostimulant treatment of negative symptoms of schizophrenia: challenges and therapeutic opportunities. Schizophr Res 2013;147:241–252. 23619055 10.1016/j.schres.2013.03.019

[51] Barch DM , Carter CS . Amphetamine improves cognitive function in medicated individuals with schizophrenia and in healthy volunteers. Schizophr Res 2005;77:43–58. 16005384 10.1016/j.schres.2004.12.019

[52] Lieberman JA , Kane JM , Alvir J . Provocative tests with psychostimulant drugs in schizophrenia. Psychopharmacology 1987;91:415–433. 2884687 10.1007/BF00216006

[53] Karatekin C , White T , Bingham C . Shared and nonshared symptoms in youth‐onset psychosis and ADHD. J Atten Disord 2010;14:121–131. 19805623 10.1177/1087054709347434 PMC3063058

[54] Ramos‐Quiroga JA , Nasillo V , Richarte V et al. Criteria and concurrent validity of DIVA 2.0: a semi‐structured diagnostic interview for adult ADHD. J Atten Disord 2016;33:S630. 10.1177/1087054716646451 27125994

